# The effect of minimally invasive or open radical antegrade modular pancreatosplenectomy on pancreatic cancer: A multicenter randomized clinical trial protocol

**DOI:** 10.3389/fonc.2022.965508

**Published:** 2022-09-15

**Authors:** Menghua Dai, Hanyu Zhang, Yinmo Yang, Dianrong Xiu, Bing Peng, Bei Sun, Feng Cao, Zheng Wu, Lei Wang, Chunhui Yuan, Hua Chen, Zheng Wang, Xiaodong Tian, Hangyan Wang, Wenjing Liu, Jianwei Xu, Qiaofei Liu, Yupei Zhao

**Affiliations:** ^1^ Department of General Surgery, Peking Union Medical College Hospital, Chinese Academy of Medical Sciences, Peking Union Medical College, Beijing, China; ^2^ Department of General Surgery, Peking University First Hospital, Beijing, China; ^3^ Department of General Surgery, Peking University Third Hospital, Beijing, China; ^4^ Department of Pancreatic Surgery, West China Hospital, Sichuan University, Chengdu, China; ^5^ Department of Pancreatic and Biliary Surgery, The First Affiliated Hospital of Harbin Medical University, Harbin, China; ^6^ Department of General Surgery, Xuan Wu Hospital, Capital Medical University, Beijing, China; ^7^ Department of Hepatobiliary Surgery, The First Affiliated Hospital of Xi’an Jiaotong University, Xi’an, China; ^8^ Department of Pancreatic Surgery, General Surgery, Qilu Hospital, Cheeloo College of Medicine, Shandong University, Jinan, China

**Keywords:** clinical trial, design, pancreatic cancer, radical antegrade modular pancreatosplenectomy, minimally invasive surgery, length of stay, protocol

## Abstract

**Background:**

Radical antegrade modular pancreatosplenectomy (RAMPS) has been proven to improve R0 resection and lymph harvest in treating patients with distal pancreatic cancer. The development of minimally invasive surgery has advantages in postoperative recovery. Therefore, minimally invasive (MI-) RAMPS may combine the advantages of both benefits to improve survival. Nevertheless, evidence to validate the safety and efficacy of MI-RAMPS is limited.

**Method/Design:**

The MIRROR trial will be the first multicenter prospective randomized clinical trial to investigate the outcome of MI-RAMPS. The hypothesis is that MI-RAMPS is superior in postoperative recovery. The primary outcome is the length of postoperative stay. Based on the hypothesis and primary outcome, the sample size is 250 patients (125 participants in each group). The trial will investigate factors related to surgical safety, short-term outcome, pathological assessment, and survival as secondary outcomes.

**Conclusion:**

This study will offer a relatively higher level of evidence to further illustrate the accessibility and benefits of MI-RAMPS for the treatment of distal pancreatic cancer.

**Clinical Trial Registration:**

Clinicaltrials.gov, NCT03770559.

## Introduction

Distal pancreatic cancer is a poorly-diagnosed disease with the highest incidence-to-mortality ratio worldwide (1, 2). The high incidence-to-mortality ratio is mainly due to delayed diagnosis, which limits treatment efficacy and options (3). Most recent consensus and guidelines recommend surgical resection if the primary tumor is resectable or borderline-resectable after neoadjuvant chemotherapy (3, 4). However, conventional distal pancreatosplenectomy (CDPS), which was recommended as one of the standard procedures for distal pancreatic cancer, has been reported to have unsatisfactory oncological outcomes in patients in recent years (5–7).

Accordingly, Strasberg et al. proposed radical antegrade modular pancreatosplenectomy (RAMPS) as one of the improved procedures for distal pancreatic cancer (8, 9). When compared with CDPS (5, 7, 10, 11), the procedure does not increase perioperative risks. Additionally, the expanded clearance of RAMPS results in better R0 resection rates and lymph node retrievals than CDPS. Therefore, compared with CDPS, RAMPS has been proven to be a safe procedure with better survival for patients with pancreatic cancer.

In the era of enhanced recovery surgery, minimally invasive surgery (MIS) has been widely accepted in the treatment of the most benign or low malignancy neoplasms of the pancreas (12). Nevertheless, the applicability of MIS in treating pancreatic cancer is controversial. The surgical outcomes and oncological safety of open and MIS procedures are of great interest among pancreatic surgeons. On the one hand, MIS is considered to result in less pain and shortened recovery time for the following anti-cancer treatments (13, 14). On the other hand, some malignant pancreatic cancer removals, such as RAMPS particularly, often have expanded surgical areas with the potential of more aggressive resection (8, 9).

In a previous study, we reported that the RAMPS cohort had a higher survival rate than the CDPS cohort (15). Subsequently, we performed a retrospective comparison between minimally invasive RAMPS (MI-RAMPS) and open RAMPS and found that MI-RAMPS is safer and has the potential advantages of faster recovery (16). Nevertheless, no prospective randomized clinical trial on the advantages of MI-RAMPS has been conducted. Therefore, the MIRROR study aims to conduct a multicenter, randomized controlled study to compare MI-RAMPS and open RAMPS (O-RAMPS) in treating patients with distal pancreatic cancer. This study will offer higher level evidence to pancreatic surgeons on the optimal use of MI-RAMPS to improve patients’ postoperative recovery and combine with neo-/adjuvant therapy for better survival.

## Method

### Design

**Figure 1:**
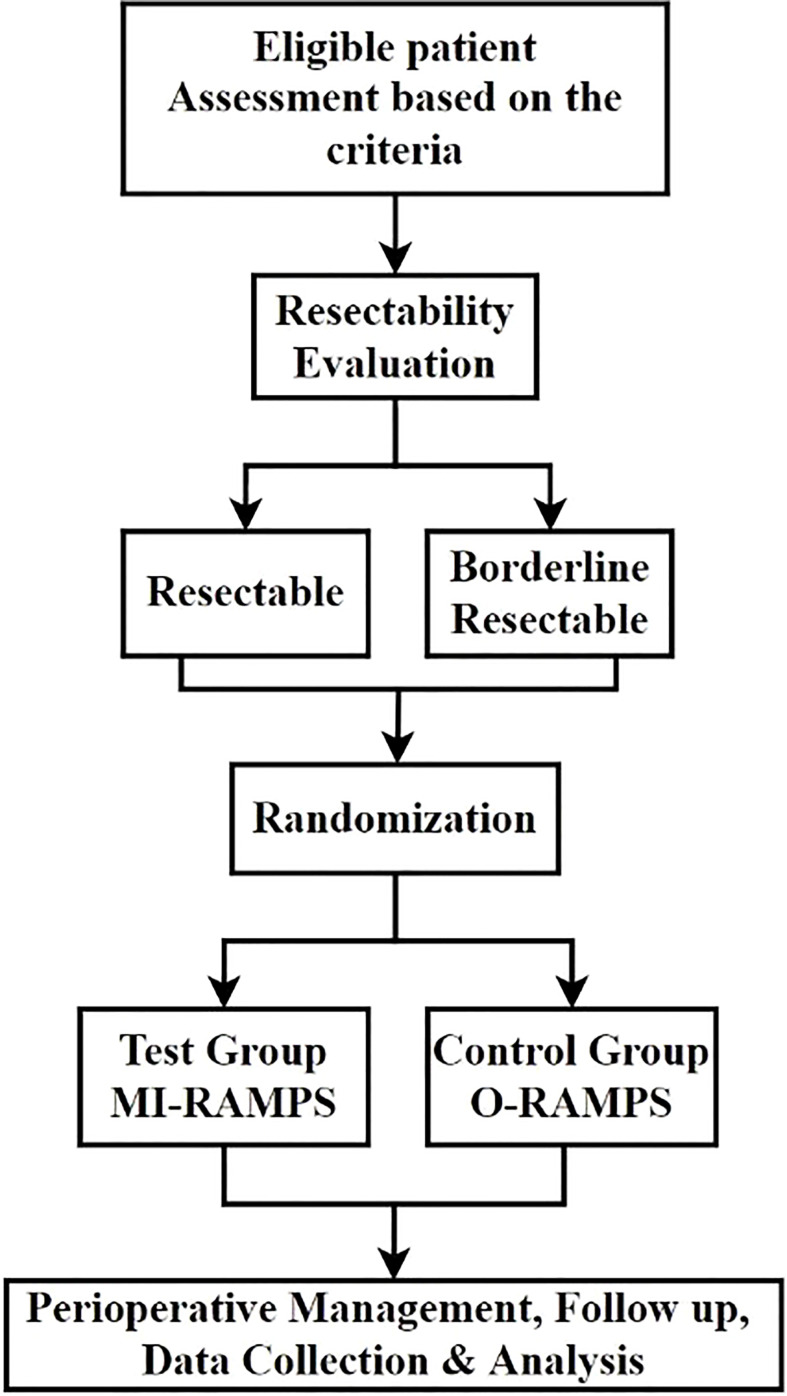
Study flowchart of MIRROR study.

The MIRROR trial is a randomized controlled, parallel-group, multicenter, superiority trial investigating and comparing the safety and effect of MI-RAMPS and OP-RAMPS for pancreatic cancer (**Figure 1**). Eligible patients will be randomly assigned to either the MI-RAMPS or O-RAMPS treatment group. This trial protocol is based on the SPIRIT guidelines and checklist (17).

### Study population

This study involves eight high-volume pancreatic surgery centers in China. Each participating center has performed >200 MI/O-RAMPS cases. All patients with suspected left-sided pancreatic cancer visiting outpatient clinics of these centers will be thoroughly evaluated according to the inclusion and exclusion criteria of the study.

### Inclusion criteria

The inclusion criteria include (i) age ≥18 years; (ii) high suspicion or pathological diagnosis of pancreatic malignancy; (iii) resectable or borderline resectable tumor before surgery, regardless of neoadjuvant chemotherapy history; and (iv) eligibility for both MI-RAMPS and O-RAMPS based on evaluation by surgeons and anesthetists before surgery.

### Exclusion criteria

The exclusion criteria include (i) suspicion or evidence of any distant metastasis or NCCN-defined unresectable arterial invasion; (ii) ASA physical status score ≥4; (iii) patient preference for a certain approach or change of willingness; and (iv) absence of malignancy based on the postoperative pathological report.

### Borderline resectable tumor

According to NCCN version 1.2020 guidelines and Isaji et al. (18), a tumor will be classified as borderline resectable if at least one of the following factors is recognized: (i) Solid tumor contact with the superior mesenteric artery ≤180° or >180° without the involvement of the aorta and intact uninvolved gastroduodenal artery; (ii) solid tumor contact with the superior mesenteric vein or portal vein of >180° or contact ≤180° with contour irregularity or thrombosis of the vein, but with suitable vessel proximal and distal to the site of involvement, allowing for safe and complete resection and vein reconstruction; (iii) carbohydrate antigen 19-9 (CA19-9) level >500 U/ml; and (iv) ECOG (19) performance status ≥2.

Although patients with borderline resectable tumors will be referred to an oncologist or multi-disciplinary team and recommended for neoadjuvant therapy, this situation will not be an independent factor for exclusion.

### Randomization

Eligible participants will be recruited from eight centers after providing written informed consent. Stratified blocked randomization between O-RAMPS and MI-RAMPS will be performed in a 1:1 ratio; Before randomization, patients will be assigned to two subgroups based on resectability: borderline resectable subgroup and resectable subgroup to perform independent stratified randomization. Patients identified as borderline resectable, as illustrated in “borderline resectable tumor,” will be assigned to borderline resectable subgroups. Otherwise, the case will be in the resectable group for randomization. The block sizes will be subjected to random variation. Randomization will be concealed from all investigators. Patients will be assigned codes by numeric randomization coding, and the study coordinator will be the only one with access to these codes. The source data will be stored digitally and kept in the central database. Randomization will be performed after the surgical plan is made and the written informed consent from patients and approval for the trial are available. Patients who rescind their decision to undergo surgery and those who do not undergo surgery will be excluded from the analysis.

### Surgical technique

The RAMPS procedure is based on a report by Strasberg et al. (8, 9). All surgeons and their surgical teams are skilled in performing this procedure. To optimize for the best outcome, surgeons can decide to perform anterior or posterior RAMPS based on their evaluation of certain clinical cases during the surgery. Meanwhile, tiny variations in lymph node dissection and necessary extended tissue or organ resection, which are not beyond the guideline, are allowed (8, 9, 20, 21). In the MI-RAMPS group, all surgical teams can choose the appropriate general laparoscopic method or robotic techniques, such as the da Vinci^®^ Surgical System, according to their preference and availability of resources.

### Conversion

Conversion is defined as any case requiring additional hand-assisted approaches, except those for trocars and specimen collection, in the MI-RAMPS group (22, 23). Based on practical scenarios, the conversion will consist of reactive conversions (such as bleeding and organ perforation) and conditional conversions (such as difficult exposure, failure to proceed, and expanded tumor evasion) (22). The details about the conversion will be recorded for future analysis. According to the principle of intention-to-treat, patients who will undergo conversion will be continually analyzed in the MI-RAMPS group.

### Blinding

The MIRROR trial is an open-label trial. However, several approaches will be applied to minimize the interference of subjective factors in the study findings. For example, patients will provide informed consent for both approaches and the study. However, they will be blinded to their specific groupings during the treatment. After the treatment, patients will not be actively informed of the specific surgical steps and procedures. The patient, however, reserves the right to know or quit anytime. Neither the pathologists involved in the postoperative evaluation nor the adjudication committee will be informed of treatment assignments.

### General treatment regimen

The strategy and protocol of the general treatment during the perioperative period for both MI-RAMPS and O-RAMPS groups are the same. They include prophylactic drainage, nutritional support, anti-infection, proton pump inhibitor use, somatostatin use, blood sugar management, pain management, deep vein thrombosis prevention, and existing disease management. Other treatments, such as interventional therapy and reoperation, will be performed, if necessary, for the management of severe complications after the surgery.

### Primary outcome

The primary outcome is the postoperative length of stay (LOS). Experienced surgeons will be responsible for the approval of discharge based on uniform criteria (24), including (i) no need for IV fluid; (ii) performance status and organ function recovery to the preoperative state; (iii) solid diet availability; (iv) no sign of infection; and (v) acceptable incision healing and pain control. However, discharge criteria do not include prophylactic drainage removal.

Patient evaluation will be recorded on case report forms (CRF) based on the observation and medical records for every round. Once the patient is eligible for discharge, the investigator will record the condition and date for the calculation of LOS, regardless of the similarity of the actual discharge date, as it may be affected by certain non-medical factors. If the evaluation for some cases is controversial, the senior surgical adjudication committee will be consulted.

### Secondary outcomes

The secondary outcomes of the MIRROR study include surgical outcomes, complications, pathological outcomes, time and rate of return to adjuvant therapy, and long-term survival.

### Surgical outcomes and complications

The details during the surgery will be recorded, including procedure type, surgical team, surgery duration, estimated blood loss, transfusion, conversion, combined vessel, and/or organ resection. To evaluate the quality of life, the VAS score, QLQ-C30, and QLQ-PAN26 will be used postoperatively.

Additionally, the rate of major complications and their detailed management will be recorded and investigated. Major complications will be defined according to the Clavien-Dindo grade III-IV classification system (25). Common complications after pancreatic surgery, such as postoperative pancreatic fistula, delayed gastric emptying, and hemorrhage, are classified based on the International Study Group on Pancreatic Surgery (ISGPS) guidelines (26–29). Only Grade B and C pancreatic fistula will be identified.

### Pathological outcomes

All specimens will be collected for lymph node sorting, incision margin marking, and labeling by surgical team members (under the supervision of seniors or operators) and then sent to two pathologists for evaluation. R0 resection rate is one of the crucial secondary factors. R0 resection is recognized when the distance between the margin and tumor is >1 mm. R0 resection will be mainly evaluated using either the transection margin or retroperitoneal margin. Multiple pathological factors, such as the number of lymph node harvests, number of positive LN, LN ratio, and status of margin, will be recorded and analyzed if detectable (30). TNM staging according to the American Joint Committee on Cancer (AJCC) classification (8th edition) will be recorded (31).

### Long-term survival

Disease-free survival (DFS) and overall survival (OS) will be used as secondary outcomes of this study. DFS is the postoperative survival period without recurrence and metastasis of the primary tumor. OS is defined as the entire length of survival after the RAMPS procedure. All related survival information, including recurrence, metastasis, and survival status, and the subsequent anti-cancer treatment information will be acquired during the postoperative follow-up. The anticipated mean follow-up period for the survival study is 24 months.

### Patient follow-up

The follow-up plan consists of out-patient clinic visits 1, 3, and 6 months after the surgery. Thereafter, patients will be followed up every six months. In case of a no-show in the clinic, the interview by phone will be conducted at every interval. Detailed information on symptoms, lab tests, medical imaging examinations (ultrasound, CT, MRI, or PET-CT), adjuvant therapy regimen, recurrence, metastasis, and survival status will be recorded at every follow-up visit.

### Data collection

All data of enrolled patients will be gathered into a central database, the Electronic Data Capture (EDC) system, based on the previous design of the CRF, which consists of baseline information, randomization result, lab test result, medical examination, surgical treatment information, peri-operative management record, and follow-up data. To secure quality and confidentiality, all data will be under surveillance by a third-party professional data management team.

### Quality and safety

All participating centers and their surgical teams are experienced in RAMPS procedures and other pancreatic surgical procedures. Each center has at least one senior surgeon who will join the surgical adjudication committee, comprising seniors from other centers, to ensure safety and procedure standards. All the senior surgeons are specialists in pancreatic surgery and will be available for assessment and consultation for difficult cases during the trial.

Regarding histopathological evaluation, all specimens, including primary tumor and resected lymph nodes, will be collected and marked (such as resection margin) by the surgical team before transfer to the pathological team. At least two expert pathologists will evaluate every case, one being a senior specialist in hepatopancreatobiliary (HPB) disease diagnosis. If the assessment is inconsistent with that of the other pathologist, another HPB pathologist will be invited for final evaluation.

### Ethics

This trial will be conducted according to the principles of the Declaration of Helsinki (32). The study protocol has been received and approved by the Institutional Review Board (IRB) of Peking Union Medical College Hospital (No. ZS-1823). Additionally, approval was obtained according to the local regulations of all participating centers. The trial has been registered on Clinicaltrials.gov (NCT03770559).

### Statistics

#### Sample size calculation

The MIRROR trial has been designed as a superiority trial. We hypothesize that patients with distal pancreatic cancer who undergo MI-RAMPS have a shorter postoperative stay than those who undergo O-RAMPS. Based on our previous experience and related data on the retrospective cohorts (15, 16), the expected superiority in the length of stay in the MI-RAMPS group is 3 ± 8 days. These factors, 5% two-sided significance level (α), 80% power (1-β), and 10% drop-off rate, were considered in calculating the sample size. Accordingly, the expected sample size has been set at 250 patients (125 patients in each group).

#### Statistical analysis

Continuous variables will be expressed as means with standard deviation or median and compared using the independent samples T-test and Wilcoxon rank test. Categorical variables will be described as percentages and compared using the Pearson Chi-square test, continuity correction, or Fisher’s exact test. Survival will be evaluated using both OS and DFS. Clinically considerable or significant variables based on univariate analysis will be included in multivariate analysis, which will be performed by Cox regression analysis. P-value <0.05 will indicate statistical significance.

## Discussion

For resectable and borderline resectable distal pancreatic cancer, one of the main goals of surgical treatment is the complete removal of the tumor and potentially involved tissues or organs, in addition to a necessary lymph node harvest. This approach is beneficial for the patient in reducing tumor burden and preventing recurrence and metastasis (3, 4). Furthermore, satisfactory lymph node dissection can improve the accuracy of TNM staging (33). In this regard, CDPS is currently considered limited by its ability to achieve appropriate oncological safety. Therefore, RAMPS surgery is widely known to improve R0 resection rate and lymph node dissection, thereby providing a better treatment strategy for distal pancreatic malignancies. Since MIS has the advantages of less injury, less pain stimulation, and faster recovery, it may shorten the postoperative recovery period of patients with pancreatic cancer to facilitate necessary postoperative adjuvant therapy (5, 13). However, the current high-level evidence-based medicine mainly focuses on benign or low-grade malignant tumors treated by minimally invasive pancreatic surgery (34, 35). For the treatment of distal pancreatic cancer, only one protocol of an ongoing multicenter randomized controlled trial, DIPLOMA, has been published (36, 37). Nevertheless, its surgical procedure for treating pancreatic cancer is mainly based on CDPS, and the research design is a non-inferiority study on R0 resection of MIS. Therefore, the ability of MI-RAMPS to promote early recovery without compromising on safety should be investigated to improve survival.

In this trial, RAMPS will be performed using either the anterior or posterior approach according to the scope of surgical resection, which is mainly selected according to the intraoperative assessment of the chief surgeon. However, we stratified resectability before randomization because of significant differences in treatment strategy and prognosis between patients with resectable and borderline resectable tumors. A common criterion for assessing resectability is vascular invasion. However, Isaji et al. (18) have proposed certain biological criteria (CA19-9) and performance status evaluation, which have been adopted in this study. Moreover, the MIRROR trial will include a strict pathological evaluation system to reflect the oncological outcomes of MI-RAMPS. Concerning the pathology report evaluation, we will follow the margin evaluation system proposed by the Japan Pancreas Society (30) and TNM staging by AJCC to evaluate the margins, lymph nodes, and suspicious invasion in multiple dimensions to ensure the diagnostic accuracy of the pathology report.

The primary outcome of this study will be the length of postoperative hospital stay. LOS could directly and better reflect the period between operation and postoperative adjuvant therapy. In this study, all participating centers and their pancreatic surgical teams are well-experienced in both O-RAMPS and MI-RAMPS, as well as general peri-operative management. Moreover, each center has assigned at least one senior pancreatic specialist to join the adjudication committee for supervision and consultation. Therefore, to a certain extent, we believe that based on accurate evaluation criteria, the results of the corresponding superiority of LOS will indicate that MI-RAMPS is beneficial for enhanced postoperative recovery. By validating this hypothesis, we expect to provide distal pancreatic cancer patients with a safe, minimally invasive way to get the tumor radical removal and receive the necessary postoperative adjuvant therapy timely for the best chance of survival.

The MIRROR study is the first multicenter prospective randomized clinical trial to investigate the safety and efficacy of MI-RAMPS surgery for pancreatic body and tail cancer. Admittedly, our study is not an international multicenter study, mainly due to concerns about large differences and deviations in the discharge time among each country’s national medical insurance policies and medical systems. Sample size estimation was based on LOS retrospective data from the principal investigation center, which may not be suitable for international trials. However, the conclusions will be beneficial to the exploration of further international studies and provide a reference for the establishment of RAMPS discharge standards and subsequent adjuvant therapy indications. Certainly, we encourage and look forward to conducting an international multi-center randomized clinical trial based on the results of this study for further investigation into the safety and efficacy of MI-RAMPS.

## Data availability statement

The original contributions presented in the study are included in the article/supplementary material. Further inquiries can be directed to the corresponding authors.

## Ethics statement

The conduction of the present trial is subject to the principles of the Declaration of Helsinki31. The study protocol has been received and approved by the Institutional Review Board (IRB) of Peking Union Medical College Hospital (No. ZS-1823). The patients/participants provided their written informed consent to participate in this study.

## Author contributions

MD conceived the study. MD and HZ contributed to the design of the study protocol. HZ wrote the draft of the manuscript. All authors contributed to the details of the study design, revision, and evaluation of the manuscript. All authors contributed to the article and approved the submitted version.

## Funding

This study is supported by the Project of Capital Health Research and Development of Special (No. 2020-1-4011), Beijing, China.

## Acknowledgments

The MIRROR study group would like to thank all participants for their understanding and willingness to participate in the study. Additionally, we appreciate all the contributions from our excellent physicians, nurses, and researchers of the MIRROR study group.

## Conflict of interest

The authors declare that the research was conducted in the absence of any commercial or financial relationships that could be construed as a potential conflict of interest.

## Publisher's note

All claims expressed in this article are solely those of the authors and do not necessarily represent those of their affiliated organizations, or those of the publisher, the editors and the reviewers. Any product that may be evaluated in this article, or claim that may be made by its manufacturer, is not guaranteed or endorsed by the publisher.
